# The influence of resveratrol and β-Hydroxy-β-methyl butyric acid supplementation alone or in combination on the development and health of the duodenum in Tibetan sheep

**DOI:** 10.3389/fmicb.2025.1612102

**Published:** 2025-07-08

**Authors:** Yu Zhang, Qiurong Ji, Linsheng Gui, Kefyalew Gebeyew, Shengzhen Hou, Zhiyou Wang, Lijuan Han, Chao Yang

**Affiliations:** ^1^Key Laboratory of Qinghai-Tibet Plateau Grazing Yak and Tibetan Sheep Animal Nutrition and Feed-Forage, Ministry of Agriculture and Rural Affairs, State Key Laboratory of Plateau Ecology and Agriculture, Qinghai University, Xining, China; ^2^College of Agriculture and Animal Husbandry, Qinghai University, Xining, China; ^3^CAS Key Laboratory for Agro-Ecological Processes in Subtropical Region, National Engineering Laboratory for Pollution Control and Waste Utilization in Livestock and Poultry Production, South-Central Experimental Station of Animal Nutrition and Feed Science in Ministry of Agriculture, Hunan Provincial Engineering Research Center for Healthy Livestock and Poultry Production, Institute of Subtropical Agriculture, The Chinese Academy of Sciences, Changsha, China

**Keywords:** antioxidant capacity, β-Hydroxy-β-methyl butyric acid, immunity, resveratrol, Tibetan sheep

## Abstract

Resveratrol (RES) and β-Hydroxy-β-methyl butyric acid (HMB) have been shown to exert antioxidant, anti-inflammatory effect as well as influence intestinal microbiota composition in monogastric animal. However, the mechanism by which RES and HMB regulate duodenal function in ruminants remains unclear. In this study, the effects of RES and HMB on the development and health of the duodenum in Tibetan sheep were investigated. A total of 120 early weaned Tibetan rams with similar initial body weight (15.5 ± 0.14 kg) were selected and randomly allocated into 4 groups. Each group received a basal diet supplemented with 1.5 g RES/d (RES group), 1.25 g HMB/d (HMB group), 1.5 g RES/d plus 1.25 g HMB/d (RES_HMB group), and without any additives (CON group). The results showed that RES and HMB supplementation significantly increased (*p* < 0.05) the villus height and the ratio of villus height to crypt depth in the duodenum. Meanwhile, the concentrations of cellulase and trypsin were increased (*p* < 0.05) when RES and HMB were supplemented in the basal diet. The RES group exhibited higher (*p* < 0.05) levels of anti-inflammatory markers (IgG, IL-6, and IL-1β), while combined supplementation of RES and HMB promoted (*p* < 0.05) the duodenal antioxidant capacity, as indicated by increased levels of GSH-Px and T-AOC. Furthermore, the RES group was characterized by a higher relative abundance of *Butyrivibrio*, while the HMB group was characterized by a higher relative abundance of *Aeromonas*, *Rummeliibacillus*, *UCG-002*, *Ralstonia* and *Stenotrophomonas* (*p* < 0.05 and LDA > 3.5). Metabolomics analysis showed that differential metabolites were significantly enriched (*p* < 0.05) in the cyanoamino acid metabolism, biosynthesis of secondary metabolites, protein digestion and absorption and ABC transporters. These results indicated that RES and HMB can be used as feed additives to maintain intestinal health by enhancing duodenal development, promoting digestive enzyme secretion, and improving antioxidant and anti-inflammatory capacity in Tibetan sheep.

## 1 Introduction

With the rapid development of the human population and economy, the demand for high-quality meat is increasing, which accelerates the transition of the livestock production system (Tufekci and Sejian, 2023). However, with the transition of the livestock production system, sheep farming is toward intensification which is characterized by a short production cycle and higher production (Soriano et al., 2024). In the current extensive sheep farming system, new problems including respiratory and gastrointestinal diseases continually occur leading to higher morbidity and mortality (Rout and Behera, 2021). To prevent respiratory and gastrointestinal diseases under intensive production systems, antibiotics are widely used in animal husbandry. However, overuse of antibiotics often leads to excessive antibiotic residues in meat products (Shao et al., 2021). Thus, announcement No. 194 of the Ministry of Agriculture and Rural Affairs of the People’s Republic of China states prohibiting the use of all growth-promoting pharmaceutical feed additives, including antibiotics in animal production effective from January 1, 2020. To ensure safe livestock and sheep production, natural and safe alternatives to antibiotics are needed to promote growth performance and maintain intestinal health.

Resveratrol (RES), a natural active polyphenol, is extracted from peanuts, grapes, pineapples, blueberries, and other plant sources (Chaplin et al., 2018; Tian and Liu, 2019). Dietary supplementation of RES can reduce gastrointestinal oxidative stress and inflammatory responses while improving lipid metabolism in animals (Zhang H. Z. et al., 2019). Furthermore, RES supplementation affects the ruminal bacterial community in calves and sheep (Ma et al., 2015; Zhang R. et al., 2019). It also improves antioxidant status and immunity by manipulating ruminal microorganisms and metabolites throughout the reproductive cycle in ewes (Li et al., 2024) As an intermediate product of the leucine metabolism, β-Hydroxy-β-methylbutyric acid (HMB) is extensively used in human nutrition and animal nutrition to promote protein synthesis (Duan et al., 2016). Studies have been demonstrated that HMB improves growth performance, antimicrobial activity, and anticatabolic and anabolic activity in monogastric animals. It also increases the concentration of growth hormone, insulin-like growth manipulating ruminal microorganisms, metabolites, and the activity of bone alkaline phosphatase (Tomaszewska et al., 2023). Furthermore, dietary HMB supplementation alters ruminal microbiota, especially by increasing the relative abundance of *Fibrobacter succinogenes* and *Ruminococcus flavefaciens* in cows. Thus, RES and HMB could act as a substitute for antibiotics in sheep farming and have no residue and pollution in meat products.

Tibetan sheep (*Ovis aries*) are a unique breed adapting to harsh environments, including hypoxia, cold, strong ultraviolet radiation, and seasonal shortages of grassland nutrition. They are mainly distributed across the Qinghai Tibetan Plateau (QTP) and its adjacent regions, with an average altitude of more than 3,000 m (Han et al., 2024). Due to the growing human population on the QTP, the grassland resources re increasingly insufficient to meet the needs of animal husbandry. To promote the sustainability and productivity of the Tibetan sheep industry, the production modes are gradually transitioning toward intensification. Supplementing diets with natural and safe feed additives is essential to promote growth performance and maintain gastrointestinal health. The duodenum, the first section of the small intestine, contains a vast number of villi and microvilli, which facilitate nutrient absorption (Moharrery et al., 2014). It is also the point at which pancreatic fluid and bile enter the digestive tract, thereby assisting the intestines in the breakdown of fats, proteins, and starches (Wang et al., 2025). The mucosal layer is composed of a high concentration of immune cells that are capable of recognizing and eradicating pathogens that penetrate the intestinal tract (Vancamelbeke and Vermeire, 2017). Duodenum plays an important role in the regulation of digestion, absorption of essential micronutrients and macronutrients, and maintaining bowel motility (Shames, 2019). The health status of the duodenum directly influences the secretion of bile and digestive enzymes, which in turn affects the feed conversion rate of sheep. However, there is little information on how dietary supplementation with RES and HMB supplementation affects duodenal function in Tibetan sheep.

In the current study, we aimed to investigate the effects of supplementation of RES and HMB, alone or in combination, in the basal diet of Tibetan sheep on duodenal digestive enzyme concentrations, morphogenesis, antioxidant capacity, immunity, microbiology, and metabolome. While previous studies have focused on the effects of additives on monogastric animals, our study focused on ruminants, particularly Tibetan sheep and provide new insights into the use of additives. Through a comprehensive study of a range of physiological and biochemical parameters, our findings not only elucidate the mechanisms by which RES and HMB regulate duodenal function, but also provide practical guidance for optimizing the nutrition and health of livestock in challenging ecological environments.

## 2 Materials and methods

### 2.1 Ethical statement

The experimental protocols of this study were performed in accordance with the guidance of the Animal Ethics Committee of Qinghai University (Permit No. QUA-2020-0709). All Tibetan sheep included in the current study were brought from a local farm (Qinghai Xiangkameiduo Animal Husbandry Co., Ltd., Qinghai, China) and the animal trial was conducted from April 2023 to August 2023.

### 2.2 Animal and experimental design

A total of 120 early weaned Tibetan rams with similar initial body weight (15.5 ± 0.14 kg) and healthy body condition were selected and randomly divided into 4 treatments. The dietary treatments were as follows: (1) fed a basal diet only (control group, CON); (2) a basal diet supplemented with 1.5 g resveratrol /d (resveratrol group, RES); (3) a basal diet supplemented with 1.25 g β-Hydroxy-β-methyl butyric acid /d (β-Hydroxy-β-methyl butyric acid group, HMB); (4) a basal diet supplemented with 1.5 g resveratrol/d plus 1.25 g β-Hydroxy-β-methyl butyric acid/d (RES_HMB). In the current study, Tibetan rams in each treatment were randomly allocated into 5 pens (with an average area of 20 m^2^) with 6 rams in each pen. The animal feeding trial lasted for 90 days following 10 days of adaptation, all rams had free access to water during the above experimental period. Lambs received their respective diets two times daily at 0830 h and 1,630 h and were fed ad libitum, the forage-to-concentrate ratio of the basal diet was 7:3. The ingredient composition and nutritional levels of the basal diet are listed in Table 1.

**Table 1:** Ingredient composition and nutritional levels of basal diet.

Item	Content (%)
Ingredient, % DM basis	
Oat hay	15.00
Corn silage	15.00
Corn	36.00
Soybean meal	1.40
Rapeseed meal	9.00
Cottonseed meal	1.40
Palm meal	17.50
NaCl	0.70
Limestone	0.70
NaHCO_3_	0.10
Premix^1^	3.20
Chemical composition	
Digestible energy, MJ/kg DM^2^	11.78
Crude protein, g/kg DM	12.69
Ether extract, g/kg DM	3.11
Neutral detergent fiber, g/kg DM	35.04
Acid detergent fiber, g/kg DM	23.88
Ca, g/kg DM	0.38
P, g/kg DM	0.17

1 Premix provided for per kilogram of diets: Cu 18 mg, Fe 66 mg, Zn 30 mg, Mn 48 mg, Se 0.36 mg, I 0.6 mg, Co 0.24 mg, VA 24,000 IU, VD 4,800 IU, VE 48 IU.

2 Digestible energy is calculated based on the ingredients of the diet and their digestible energy content.

### 2.3 Sample collection

Upon completion of the trial, 24 Tibetan rams (*n* = 6 per group, 2 rams were selected from each pen in the same group) were slaughtered by bleeding the jugular vein by a registered veterinarian after 15 h of fasting. Following the abdominal cavity dissection, the junction of the abomasum and duodenum, and the junction of the duodenum and jejunum were ligated using a cotton cord. Subsequently, the duodenal contents were collected into 15 mL germ-free centrifuge tubes (Corning®, NY, United States), transferred into liquid nitrogen for snap-frozen and then stored at −80°C for microbial genomic DNA isolation and metabolomics analysis. Afterward, the duodenal tissues were washed with ice-cold 0.9% saline solution three times to remove residual digesta, then chipped into small pieces and placed in 4% paraformaldehyde for histological analysis, the remaining tissues were collected into a sterile sample bag (B01064, Whirl-Pak^®^, WI, United States) and frozen in liquid nitrogen and stored at −80°C for determining immune indexes and antioxidant capacity.

### 2.4 Determination of digestive enzyme

Duodenal content sample (approximately 1 g) was weighed and homogenized in ice-cold phosphate buffer saline (pH 7.4, Solarbio LIFE SCIENCES, Beijing, China) using a handheld glass homogenizer for 5 min and vortex mixer (Vortex-Genie G560E, Scientific Industries, New York, United States) was used to suspend the homogenate for three times during this period. Then, the homogenate was centrifuged at 12,000 rpm for 15 min at 4°C, and the supernatant was collected in a 1.5 mL tube for assays of the total protein and digestive enzyme. The levels of α-amylase, lipase, trypsin, cellulase, and chymotrypsin were detected using a commercial kit according to the manufacturer’s protocols without any modification (Jiangsu Meimian industrial Co., Ltd., Yancheng, China).

### 2.5 Duodenal morphology

Following fixed in 4% paraformaldehyde for 48 h, the duodenal tissue sample was dehydrated in gradient ethanol, and finally embedded in the paraffin wax. Three sections for each sample were sliced, installed on glass slides and stained with eosin and hematoxylin. Three random straightest villi and their accompanying crypts of each slide were selected to measure the villus height, crypt depth, mucosal thickness and muscular thickness using a microscope (BX51, Olympus, Tokyo, Japan) coupled with Imagingeproplus 6.0 analysis software. The V/C value (ratio of villus height to crypt depth) was calculated.

### 2.6 Measurement of immune indexes and antioxidant capacity

Approximately 200 mg duodenal tissue was homogenized in ice-cold saline using a handheld glass homogenizer for 3–5 min and vortex mixer (Vortex-Genie G560E, Scientific Industries, New York, United States) was used to suspend the homogenate for 3 times during this period. Subsequently, the homogenate was centrifuged at 12,000 rpm for 10 min at 4°C, supernatant was collected and used for the determination of cytokine concentration and antioxidant capacity. The concentrations of immunoglobulin (IgA, IgG, IgM) and cytokines including interleukin-1β (IL-1β), interleukin-6 (IL-6) and tumor necrosis factor-α (TNF-α) were determined using sheep specific ELISA kits (Jiangsu Meimian industrial Co., Ltd., Yancheng, China) according to the manufacturer’s guidance.

### 2.7 Changes of duodenal microbiota

Genomic DNA from 200 mg of each duodenal digesta was extracted using the E. Z. N. A.^®^Stool DNA Kit (D4015, Omega, United States) according to manufacturer’s instructions. The concentration and quality of the isolated DNA were determined using a Nanodrop 2000 spectrophotometer (Thermo Fisher Scientific, United States) and 2% agarose gel electrophoresis. The eligible DNA was used for PCR amplify with the primers (341F-805R) of hypervariable V3-V4 region in the bacterial 16 s rRNA gene. The amplicon libraries were sequenced on Illumina NovaSeq PE250 platform (CA, United States) after that were purified by AMPure XT beads (Beckman Coulter Genomics, Danvers, MA, United States).

Raw sequence data generated from the platform were analyzed using the QIIME2 pipeline (Bolyen et al., 2019). Briefly, paired-end reads were assigned to samples according their unique barcode and merged by using FLASH (Magoč and Salzberg, 2011). Fqtrim (version 0.94) was employed to perform quality control with specific filtering conditions to obtain high-quality reads, and the chimeric sequences were removed using Vsearch (version 2.3.4) (Rognes et al., 2016). The remain sequences were clustered into features, the feature table and denoised feature sequences which also be called amplicon sequence variants (ASVs) were obtained using the DADA2 package (Callahan et al., 2016). Then, the feature sequences were annotated with the SILVA database (version 138) for each representative sequence, and feature abundance was normalized using the relative abundance of each sample at the different taxonomic levels (Quast et al., 2013). The alpha diversity indices of ACE, Chao1, Shannon and Simpson and values of beta diversity were calculated by QIIME2 (Hall and Beiko, 2018). The linear discriminant analysis (LDA) effect size tool (LEfSe) was applied to analyze the effect size of species that contributed to the differences between the samples using the Wilcoxon test with *p* < 0.05 and LDA score > 3.5. To investigate changes in the molecular function of duodenal microbiota triggered by RES or HMB supplementation, we employed PICRUSt2 to predict the metagenomic contribution of identified microbial communities from the KEGG pathways (Douglas et al., 2020). The STAMP software (version 2.1.3) was employed to identify the different pathways between the CON and RES, CON and HMB, CON and RES_HMB groups using Welch’s t-test, and *p*-value adjusted by the Benjamini and Hochberg method (Parks et al., 2014).

### 2.8 Untargeted metabolomics analysis of duodenal digesta

Metabolites from 100 mg of duodenal contents were extracted with 1.0 mL 70% aqueous methanol at 4°C overnight. Subsequently, the mixture was centrifuged centrifugation at 10,000 *g* for 10 min, the extracts were absorbed and filtrated before LC–MS analysis. Non-targeted metabolomics was performed using an LC ESI MS/MS system (HPLC, Shim pack UFLC SHI MADZU CBM 30 A system; MS, Applied Biosystems 6500 Q TRAP). A preheated ACQUITY UPLC HSS T3 C18 column (1.8 μm, 2.1 mm × 100 mm, Waters, Massachusetts, United States) was used for chromatographic separation with the following gradient program: 95:5 V/V at 0 min, 5: 95 V/V at 11.0 min, 5:95 V/V at 12.0 min, 95:5 V/V at 12.1 min, 95: 5 V/V at 15.0 min; flow rate 0.40 mL/min; temperature 40°C; injection volume 2 μL. The effluent was alternatively connected to an ESI triple quadrupole linear ion trap (Q TRAP)-MS.

Raw data files were analyzed using Compound Discoverer 3.1 (CD3.1, Thermo Fisher) to perform peak alignment, peak picking, and quantitation for each metabolite (Yang et al., 2025). The metabolites were annotated with the MS2 internal database (BiotreeDB) with a cutoff value of 0.3 (Zhang et al., 2022). Orthogonal partial least squares discriminant analysis (OPLS-DA) was conducted to evaluate the metabolic changes among four groups using SIMCA-P software [version 14.1, Umetrics AB, Umea, Sweden, (Bylesjo et al., 2006)]. The differential metabolites were filtered using the variable importance in projection (VIP) generated in OPLS-DA, adjusted *p*-value, and fold change obtained in statistical analysis (VIP > 1, adjusted *p* < 0.05 and |fold change| > 2). Differential metabolites were conducted functional enrichment analysis using the Kyoto Encyclopedia of Genes and Genomes database (KEGG, Ogata et al., 1999; Kanehisa and Goto, 2000) and pathways on level 3 underwent variance analysis in which *p* < 0.05 was considered as a significant difference.

### 2.9 Weighted correlation network analysis

To investigate the link between phenotypes and duodenal metabolome, weighted correlation network analysis (WGCNA) packages in R software (Version 3.4.4) was employed to filter key metabolites. The metabolites detected in duodenal digesta samples from all experimental sheep were used in WGCNA analysis. Before co-expression network construction, we used pickSoft Threshold function to calculate the soft power. In the current study, we used power value 16 to construct co-expression network and selected 0.25 as mergeCutHeight parameter and merged different modules with similarity more than 70%. Module detection function was conducted with the following parameters: minBlockSize of 50, maxModuleSize of 20 and reassign threshold of 0.1. Pearson’s correlation analysis was performed to evaluate the relationship between metabolite modules and phenotypes and modules with |*r*| > 0.50 and *p* < 0.05 were defined as significant and used for downstream functional analysis.

### 2.10 Statistical analysis

Data on duodenal morphology, antioxidant capacity, immune response and digestive enzyme concentrations in duodenal contents were subjected to a one-way analysis of variance (ANOVA) procedure after checked for normality and homogeneity of variance in the SPSS software (version 25.0, SPSS Inc., Chicago, IL, United States). The difference among the four groups was evaluated using Tukey *post hoc* tests, and differences were considered statistically significant at *p* < 0.05. The results were presented as Means ± standard errors of the mean (SEM).

## 3 Results

### 3.1 Duodenal morphology

**Figure 1:**
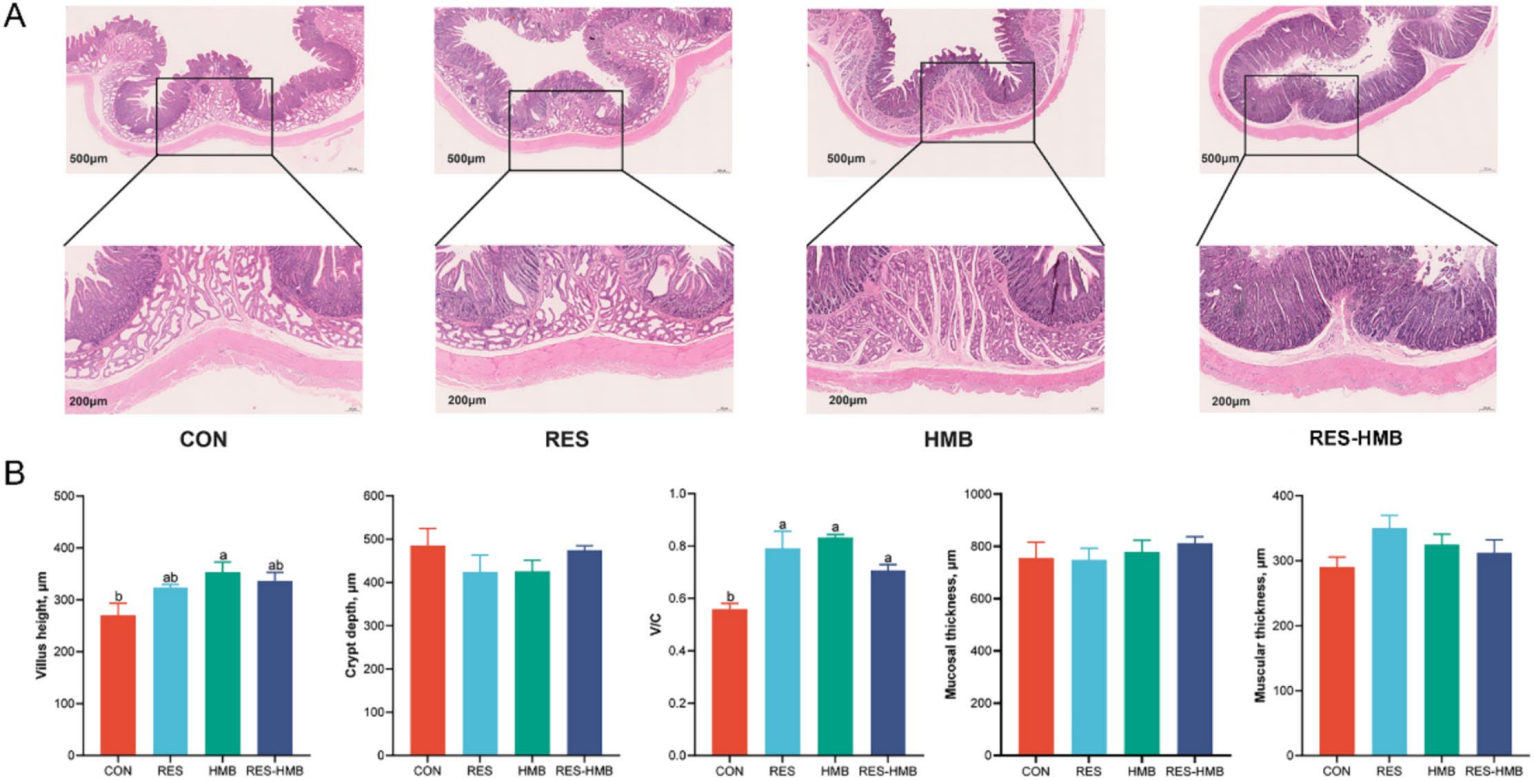
Effects of resveratrol and β-Hydroxy-β-methyl butyric acid supplementation on duodenal morphology in Tibetan sheep. **(A)** Typical histological views of duodenal slide stained with hematoxylin–eosin (original magnification 500 × and 200 × μm). **(B)** Histogram of micromorphological indicators of duodenum. V/C: villus height/crypt depth. Column with different superscript letters indicates significant differences (*p* < 0.05).

The effects of supplementation of RES and HMB, either alone or in combination on duodenal morphology are shown in Figure 1. The villus height of Tibetan sheep In the HMB group was higher (*p* = 0.015) than that in the CON group. The ratio of villus height to crypt depth (V/C) was lower (*p* = 0.001, *p* < 0.001, and *p* = 0.042, respectively) in the CON group compared to the RES, HMB, and RES-HMB groups.

### 3.2 Digestive enzyme of duodenal contents

As shown in Table 2, the inclusion of RES and HMB, either alone or in combination, in the basal diet increased (*p* < 0.05) the concentrations of cellulase and trypsin in the duodenal contents. Compared to the CON group, the HMB and RES_HMB groups exhibited a significant increase (*p* < 0.001 and *p* = 0.006) in the concentration of cellulase. Meanwhile, the concentration of trypsin in the CON group was lower (*p* < 0.001, *p* = 0.005, and *p* < 0.001, respectively) than that in the RES, HMB, and RES_HMB groups.

**Table 2:** Effects of resveratrol and β-Hydroxy-β-methyl butyric acid supplementation on the concentrations of duodenal digestive enzyme in Tibetan sheep.

Item	Group				SEM	*P*-value
	CON	RES	HMB	RES_HMB		
Lipase, ng/mg protein	216.5	212.10	241.42	230.84	6.94	0.445
Cellulase, ng/mg protein	84.99^b^	107.01^ab^	128.02^a^	118.14^a^	4.43	0.001
α-amylase, μmol/mg protein	263.44	372.81	292.95	308.58	15.86	0.084
Trypsin, ng/mg protein	56.01^b^	128.90^a^	114.80^a^	146.40^a^	9.17	<0.001
Chymotrypsin, ng/mg protein	172.18	179.73	176.55	172.05	3.64	0.871

^a,b^ Means of a row with no common superscript are significantly different (*p* < 0.05).

### 3.3 Immune indices and antioxidant capacity of duodenal tissues

The levels of immune indices of duodenal tissue are presented in Table 3. Supplementation of RES significantly elevated (*p* = 0.028 and *p* = 0.012) the levels of IgG and IL-6. Compared to the CON group, RES and RES_HMB groups exhibited a significant decrease (*p* < 0.001 and *p* = 0.006) in the level of IL-1β.

**Table 3:** Effects of RES and HMB supplementation on the levels of immune indices in duodenal tissues of Tibetan sheep.

Item	Group				SEM	*P*-value
	CON	RES	HMB	RES_HMB		
IgA, μg/g protein	1.23	2.30	1.48	1.53	0.18	0.166
IgG, μg/mg protein	584.63^b^	760.50^a^	695.74^ab^	720.74^ab^	23.29	0.036
IgM, μg/mg protein	8.56	13.16	14.32	9.78	1.20	0.288
IL-1β, ng/mg protein	28.78^a^	15.00^c^	24.53^ab^	16.35^bc^	1.56	0.001
IL-6, ng/mg protein	43.51^b^	62.12^a^	50.25^ab^	56.04^ab^	2.28	0.016
TNF-α, ng/mg protein	152.69	114.91	156.47	145.97	14.22	0.119

^a,b,c^ Means of a row with no common superscript are significantly different (*p* < 0.05).

The antioxidant capacity of duodenum in Tibetan sheep is shown in Table 4. The level of GSH-Px was higher (*p* < 0.001 and *p* = 0.013) in the RES_HMB group than that in the CON and RES groups, whereas the HMB group had a higher (*p* = 0.001) level of GSH-Px than that in the CON group. The T-AOC level of the duodenum in the RES_HMB group was higher (*p* = 0.020) than the CON group.

**Table 4:** Effects of RES and HMB supplementation on the antioxidant capacity of duodenum in Tibetan sheep.

Item	Group				SEM	*P*-value
	CON	RES	HMB	RES_HMB		
GSH-Px, pmol/g protein	31.77^c^	39.36^bc^	48.66^ab^	51.43^a^	1.99	<0.001
SOD, pg./mg protein	103.30	116.40	128.47	147.18	6.37	0.082
T-AOC, U/mg protein	6.26^b^	8.31^ab^	7.42^ab^	8.86^a^	0.34	0.023
CAT, ng/g protein	206.96	219.09	226.60	262.06	7.83	0.064
MDA, pg./mg protein	7.74	8.59	8.03	8.27	0.38	0.897

^a,b,c^ Means of a row with no common superscript are significantly different (*p* < 0.05).

### 3.4 Alterations of duodenal microbiota

**Figure 2:**
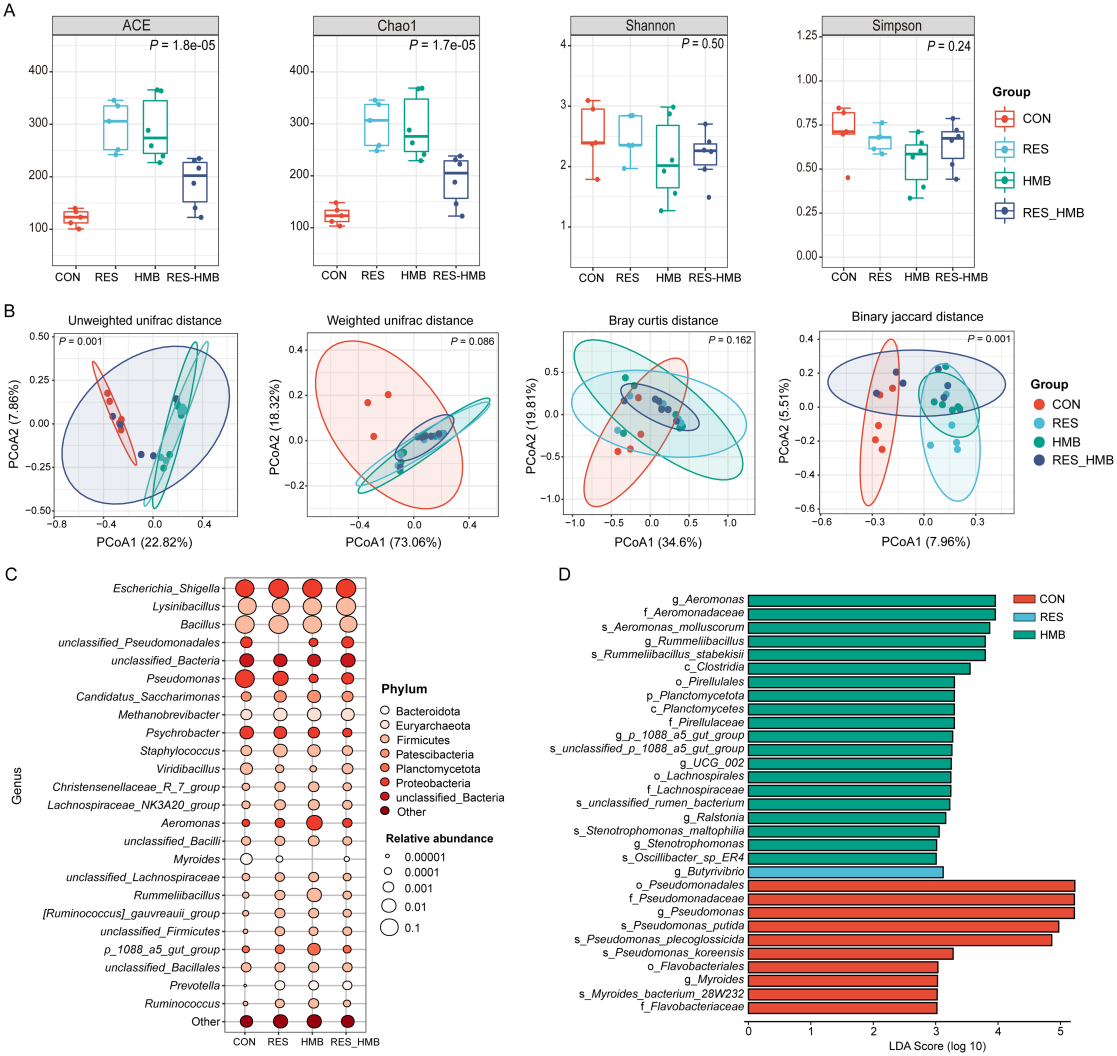
Effects of resveratrol and β-Hydroxy-β-methyl butyric acid supplementation on duodenal microbiota in Tibetan sheep. **(A)** Boxplot of alpha diversity index in duodenal microbiota. **(B)** Principal coordinate analysis (PCoA) of bacterial communities and Anosim analysis in duodenal microbiota. **(C)** Bubble plot of Top 25 bacteria on the genus level in duodenal microbiota. **(D)** Histogram of the linear discriminant analysis (LDA) effect among the four groups, and the LDA score (log10) > 3 were shown.

To investigate the effects of RES and HMB supplementation on duodenal microbiota, 16 s rDNA sequencing was employed to evaluate the diversity and bacteria composition in Tibetan sheep. A total of 2,830,900 raw reads were generated from 22 duodenal digestive samples. Two samples (one from the CON group and the other from the RES group) did not meet the requirements for library construction. After quality control, 2,354,780 clean reads were obtained, with an average of 107,035 clean reads per sample. The rarefaction curve confirmed that the sequencing data coverage was adequate to describe the duodenal bacterial composition of growing lambs (Supplementary Figure S1). The Chao1 and ACE indexes were used to assess the microbiota richness, and the Shannon and Simpson indexes were used to measure species diversity. Significant differences (*p* < 0.05) in bacterial community richness were observed among the groups (Figure 2A). In detail, the Chao1 and ACE indexes were higher (*p* < 0.05) in the RES and HMB groups than in the CON group.

**Figure 3:**
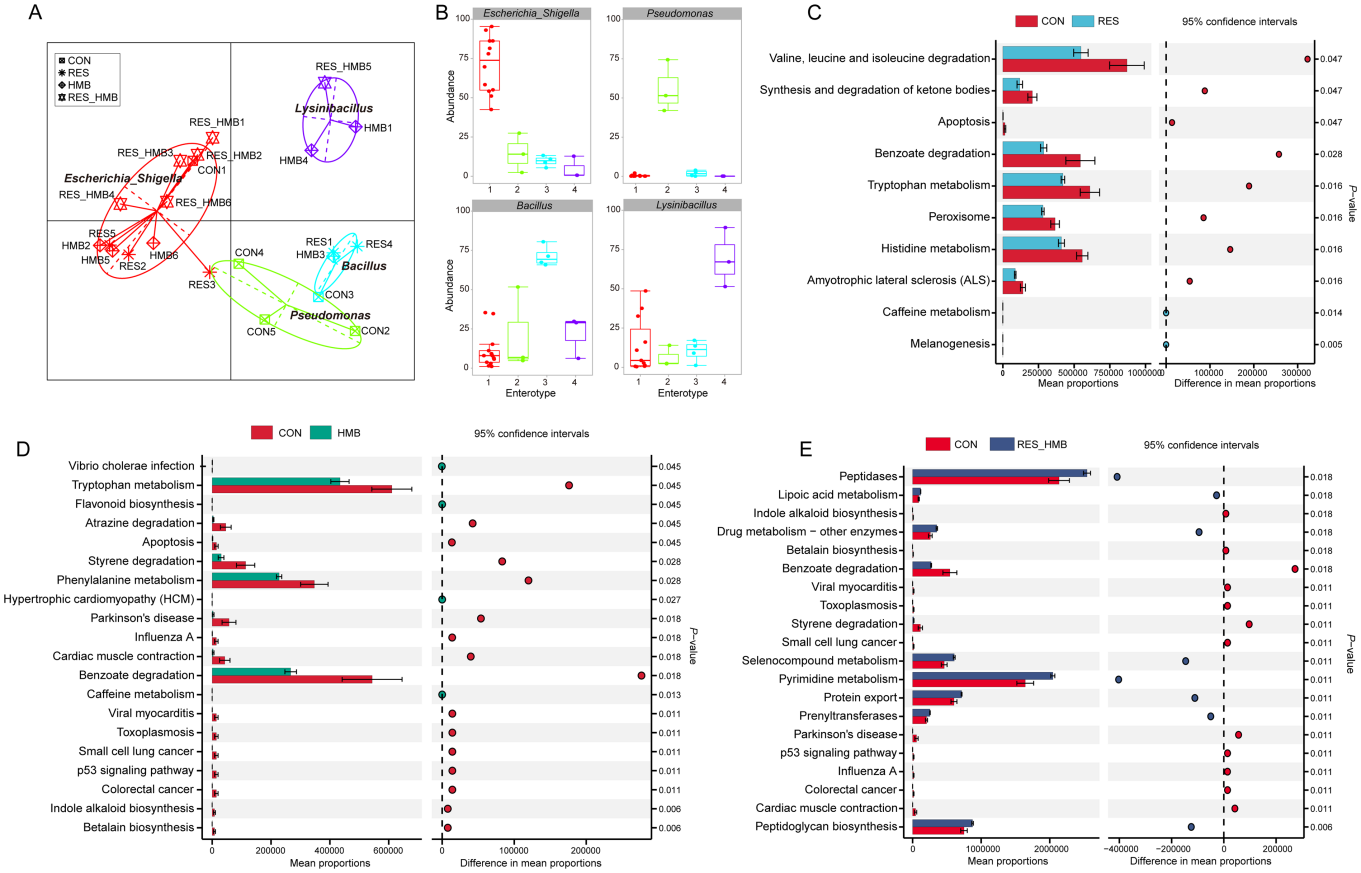
Effects of resveratrol and β-Hydroxy-β-methyl butyric acid supplementation on the enterotype and metagenomic function of duodenal microbiota in Tibetan sheep. Gut microbiome enterotype **(A)** and bacterial taxa in each enterotype **(B)**. KEGG enrichment analysis of duodenal microbiota metagenomic function in the comparison of CON vs. RES group **(C)**, CON vs. HMB group **(D)** and CON vs. RES_HMB group **(E)**.

Principal coordinate analysis (PCoA) coupled with Anosim analysis was conducted to evaluate the differences in duodenal microbiota. The PCoA plot revealed a distinct clusstering among four groups based on unweighted UniFrac (*p* = 0.001) and Binary jaccard distance (*p* = 0.001; Figure 2B). Taxonomic analysis revealed a total of 23 phyla across all groups, 7 phyla exhibiting an average relative abundance of more than 0.1% (Supplementary Figure S2). At the genus level, 24 genera were identified across all groups with an average relative abundance of more than 0.1% (Figure 2C). The LEfSe analysis at the genus level showed that the CON group was characterized by higher relative abundances of *Pseudomonas* and *Myroides*. The RES group was characterized by higher relative abundance of *Butyrivibrio*, while the HMB group was characterized by higher relative abundances of *Aeromonas*, *Rummeliibacillus*, *p_1088_a5_gut_group*, *UCG-002*, *Ralstonia* and *Stenotrophomonas* (*p* < 0.05 and LDA > 3.5; Figure 2D). To further investigate the microbial characteristics among four groups, we conducted enterotypes analysis to reveal the cluster of bacteria in each sample. A total of 4 enterotypes were identified in this study (Figures 3A,B): *Escherichia_Shigella* (enterotype 1), *Pseudomonas* (enterotype 2), *Bacillus* (enterotype 3) and *Lysomobacillus* (enterotype 4). In particular, *Escherichia_Shigella* (enterotype 1) was the main enterotype and 12 duodenal samples were enriched in enterotype 1.

### 3.5 Predicted the metagenomic metabolic function of microbiota in the duodenal digesta

A total of 266 functional pathways on KEGG level 3 were detected. Among these, 10 pathways (3.76% of the total) showed a significant difference between CON and RES groups. In detail, the CON group had a higher abundance of gene families involved in valine, leucine and isoleucine degradation, synthesis and degradation of ketone bodies, benzoate degradation, tryptophan metabolism, peroxisome, histidine metabolism and apoptosis (*p* < 0.05; Figure 3C). Compared to the HMB group, the CON group had a higher abundance of gene families associated with betalain biosynthesis, indole alkaloid biosynthesis, apoptosis, atrazine degradation, benzoate degradation, p53 signaling pathway, phenylalanine metabolism, styrene degradation and tryptophan metabolism (*p* < 0.05). Conversely, the HMB group was enriched in caffeine metabolism and flavonoid biosynthesis (*p* < 0.05; Figure 3D). As shown in Figure 3E, relative abundances of 37 pathways were changed with RES and HMB combined supplementation. Specifically, pathways such as peptidoglycan biosynthesis, tetracycline biosynthesis, steroid biosynthesis, selenocompound metabolism, RNA polymerase, ribosome, pyrimidine metabolism, protein export, prenyltransferases, peptidases, novobiocin biosynthesis, lipoic acid metabolism, glycolysis/gluconeogenesis, electron transfer carriers, dioxin degradation, carbon fixation in photosynthetic organisms, benzoate degradation, aminoacyl-tRNA biosynthesis and amino acid metabolism showed higher abundance in RES_HMB group (*p* < 0.05). Conversely, tyrosine metabolism, styrene degradation, polycyclic aromatic hydrocarbon degradation, phenylalanine metabolism, p53 signaling pathway, limonene and pinene degradation, indole alkaloid biosynthesis, betalain biosynthesis and apoptosis were more abundant in the CON group.

### 3.6 Untargeted metabolomics analysis of duodenal digesta

**Figure 4:**
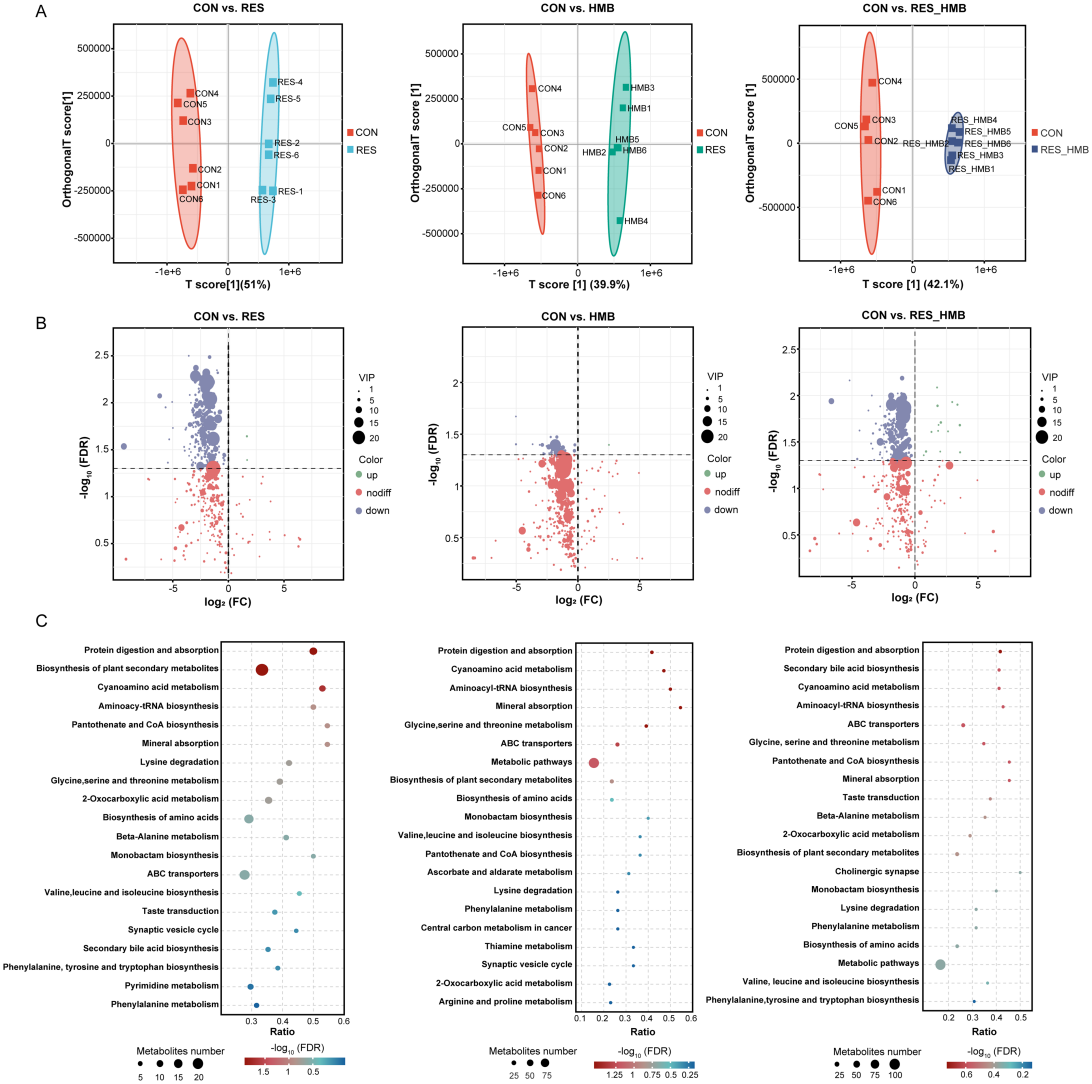
Effects of resveratrol and β-Hydroxy-β-methyl butyric acid supplementation on metabolomics of duodenal digesta in Tibetan sheep. **(A)** Scatter plots of the OPLS-DA model based on all metabolites in the duodenal digesta between the CON and RES group, CON and HMB group, and CON and RES_HMB group. **(B)** Volcano plot of differential metabolites (VIP > 1, adjusted *p* < 0.05 and |fold change| > 2) in the duodenal digesta. **(C)** The enrichment of KEGG metabolic pathways of differential metabolites in the duodenal digesta of Tibetan sheep.

The OPLS-DA model was used to evaluate the differences in metabolites among four groups. The metabolome of duodenal digesta between the CON and RES groups, CON and HMB groups, and CON and RES_HMB groups showed significantly different metabolite compositions, indicating that metabolite profiles of the duodenal digesta samples were distinct across four groups (Figure 4A). A total of 16,604 metabolites were observed in the duodenal digesta metabolome under both positive and negative ion mode, in which 3,923 metabolites were matched to the MS2 internal database (BiotreeDB). After *t*-test and VIP filtering based on the relative concentrations of metabolites, 312, 47 and 216 differential metabolites (VIP > 1, adjusted *p* < 0.05 and |fold change| > 2) were identified in the comparison of CON vs. RES, CON vs. HMB and CON vs. RES_HMB, respectively (Figure 4B). Between the CON and RES groups, 2 differential metabolites were upregulated, while 310 differential metabolites were downregulated in the RES group. We detected that only 1 differential metabolite was upregulated, while 46 differential metabolites were downregulated in the HMB group. Furthermore, 12 differential metabolites were upregulated, while 204 differential metabolites were downregulated in the RES_HMB group.

Subsequently, we conducted a KEGG enrichment analysis to reveal the biological function of differential metabolites. A total of 120 differential metabolites could map to KEGG pathways at level 3 between the CON and RES groups. These metabolites were significantly enriched (*p* < 0.05) in the following pathways: cyanoamino metabolism, protein digestion and absorption, aminoacyl-tRNA biosynthesis, mineral absorption, biosynthesis of plant secondary metabolites, glycine, serine and threonine metabolism, lysine degradation, taste transduction, biosynthesis of amino acids, phenylalanine metabolism, monobactam biosynthesis and pyrimidine metabolism (Figure 4C). In comparison between the CON and HMB groups, the differential metabolites were significantly enriched (*p* < 0.05) in Taurine and hypotaurine metabolism, secondary bile acid biosynthesis and lysine degradation (Figure 4C). As illustrated in Figure 4C, the differential metabolites between the CON and RES_HMB groups were significantly enriched (*p* < 0.05) in secondary bile acid biosynthesis, taste transduction and protein digestion and absorption.

### 3.7 Weighted correlation network analysis of duodenal metabolomics and phenotype

**Figure 5:**
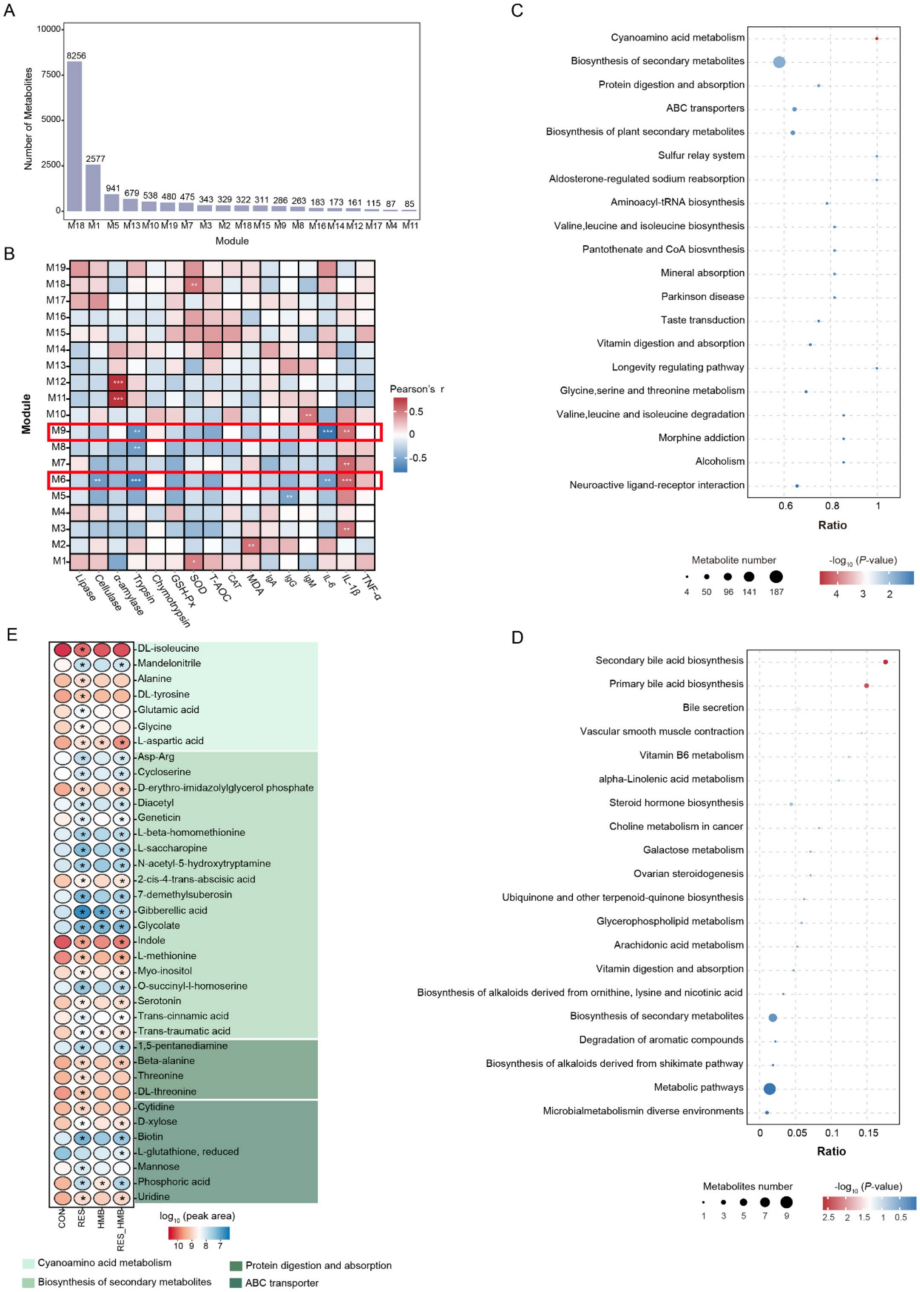
Weighted correlation network construction and key metabolites screening of duodenal digesta in Tibetan sheep. **(A)** Number of metabolites of M1 to M19 module which based on a weighted gene co-expression network analysis. **(B)** Relationship between metabolite modules (gene modules are defined as M1-M19) and phenotypic values. The enrichment of KEGG metabolic pathways of metabolites in the M6 **(C)** and M9 **(D)** module. **(E)** Differential metabolites of significantly enriched KEGG pathways in the M6 and M9 module.

To integrate the metabolomics datawith phenotypic traits, WGCNA was performed to filter key metabolites. A total of 16,604 metabolites (average peak area > 1 × 10^5^ in all samples) in duodenal digesta were clustered into 19 metabolite modules and defined as M1 to M19 modules. The number of metabolites in each module is presented in Figure 5A. The Pearson’s correlation analysis results between modules and phenotypic traits showed that the metabolites in the M6 module (8,256 metabolites, 49.72% of total metabolites) and M9 module (286 metabolites, 1.72% of total metabolites) were significantly correlated (|*r*| > 0.50 and *p* < 0.05) with phenotypic traits (Figure 5B). In detail, the metabolites in the M6 module were positively correlated (*r* = 0.63, *p* < 0.001) with IL-1β, while the above metabolites were negatively correlated with cellulase (*r* = −0.57, *p* = 0.003), trypsin (*r* = −0.72, *p* < 0.001) and IL-6 (*r* = −0.56, *p* = 0.004). The results of the KEGG enrichment analysis showed that 514 metabolites (6.23%) in the M6 module were annotated against the KEGG database. These metabolites were significantly enriched in the cyanoamino acid metabolism (3.31%), biosynthesis of secondary metabolites (36.38%), protein digestion and absorption (3.50%), ABC transporters (8.17%), biosynthesis of plant secondary metabolites (8.95%), sulfur relay system (0.97%), aldosterone-regulated sodium reabsorption (0.97%), aminoacyl-tRNA biosynthesis (2.14%), valine, leucine and isoleucine biosynthesis (1.75%), pantothenate and CoA biosynthesis (1.75%) and mineral absorption (1.75%, *p* < 0.05, Figure 5C).

A total of 16 metabolites (5.59%) in the M9 module were positively correlated (*r* = 0.57, *p* = 0.003) with IL-1β and negatively correlated with trypsin (*r* = −0.62, *p* = 0.001) and IL-6 (*r* = −0.8, *p* < 0.001, Figure 5B). After mapping these metabolites to the KEGG database, the results showed that 3 metabolites (18.75%) were significantly enriched (*p* < 0.05) in secondary bile acid biosynthesis and primary bile acid biosynthesis (Figure 5D).

### 3.8 Key metabolites screening based on weighted correlation network analysis

As mentioned above, metabolites in the M6 module were significantly enriched (*p* < 0.05) in 11 pathways. Among them, we observed 37 differential metabolites (VIP > 1, adjusted *p* < 0.05 and |log_2_(fold change) | ≥ 1) that were enriched in cyanoamino acid metabolism, biosynthesis of secondary metabolites, protein digestion and absorption and ABC transporters, respectively (Figure 5E). Specifically, 5 differential metabolites (L-aspartic acid, gibberellic acid, glycolate, trans-traumatic acid and phosphoric acid) were downregulated of the comparisons of RES vs. CON, HMB vs. CON and RES_HMB vs. CON groups. Additionally, 22 differential metabolites were downregulated in the comparisons of RES vs. CON and RES_HMB vs. CON groups. In addition, 9 differential metabolites (DL-isoleucine, alanine, DL-tyrosine, glutamic acid, glycine, threonine, DL-threonine, cytidine and mannose) were only observed in RES vs. CON and 1 differential metabolite (L-glutathione, reduced) were observed in RES_HMB vs. CON.

In the M9 module, 2 differential metabolites (glycochenodeoxycholate and glycocholic acid) were obtained in the comparison of RES_HMB vs. CON and RES vs. CON groups, respectively. These metabolites were enriched in enriched in secondary bile acid biosynthesis and primary bile acid biosynthesis.

## 4 Discussion

Under the intensive production system, animals are always susceptible to various diseases, especially gastrointestinal diseases. These disorders can disrupt nutrient utilization, impair the gastrointestinal microbiota function, and reduce the absorptive and barrier capacities of the gut epithelium, eventually leading to inflammatory responses (Plaizier et al., 2018). Thus, it is necessary to find a functional additive to maintain efficient production and intestinal health of ruminants.

Previous studies demonstrated that resveratrol exerts growth performance improvement, regulation of meat quality and fat deposition, antioxidant and anti-inflammatory in animal husbandry (Meng et al., 2023). β-Hydroxy-β-methyl butyric acid plays an important role in protein synthesis, lipid metabolism, immunological regulation and antioxidant capacity (Holeček, 2017). Except for this, dietary resveratrol supplementation enhanced the nutrient digestibility of dry matter, organic matter, neutral detergent fiber, acid detergent fiber and nitrogen in sheep (Chen et al., 2015), which suggested that dietary RES promoted nutrient digestibility but how RES affects nutrient digestibility is unknown. In the current, RES supplementation significantly promoted the concentration of trypsin in the duodenum, which may indicate dietary RES stimulates the secretion of digestive enzymes. Furthermore, HMB supplementation or RES and HMB combined supplementation significantly enhanced the concentrations of trypsin and cellulase, which was coincident with previous studies that demonstrated dietary HMB or RES and HMB combined supplementation elevated the levels of trypsin and cellulase of jejunum and ileum in Tibetan sheep (Gan et al., 2024; Ji et al., 2024), indicating RES, HMB or RES and HMB combined supplementation enhances the activity of digestive enzymes to promote the nutrient digestibility.

The function of the small intestine depends on the development of intestinal morphology, as the development of the villus largely affects the absorption of dietary nutrients (Uni, 2006). Dietary RES administration significantly enhanced jejunal villus height during the post-weaning phase in porcine models (Chen et al., 2021). HMB supplementation increased the jejunal villus height while decreasing the crypt depth of the ileum in weaned piglets (Zheng et al., 2020). In ruminants, HMB supplementation improves the development of villus and reduces the ratio of villus height to crypt depth (V/C) in the ileum (Gan et al., 2024). Similarly, HMB supplementation increased both the villus height and V/C in the duodenum, meanwhile, RES and RES combined with HMB supplementation improved the V/C ratio in the current study. The superior development of intestinal mucosal structure is essential for the efficient absorption of dietary nutrients, as villus height affects the surface area, thereby altering the nutrient absorption efficiency (Mir et al., 1987). V/C is also a representative indicator that is always used to assess the degree of nutrient absorption (Fan et al., 2022). These results indicate that RES, HMB, and RES coupled with HMB supplementation promote the development of the small intestine, especially increasing the villus height and V/C in Tibetan sheep, which may facilitate the absorption of dietary nutrients.

The digestion and absorption of dietary nutrients rely on the health status of the intestine, and intestinal health is mainly affected by the structure of intestinal microbiota and oxidative stress levels (Tomasello et al., 2016). Disruption of gut microbiota and accumulation of massive oxygen radicals caused by external factors (pathogens, harmful metabolites, heat or cold stress and drastic changes in diets) may induce intestinal inflammation response (Karl et al., 2018). Previous studies have demonstrated that RES is capable of scavenging a variety of reactive oxygen species (ROS), reactive nitrogen species (RNS), and secondary organic radicals. It achieves this through mechanisms involving hydrogen atom transfer, sequential proton loss and electron transfer, thereby safeguarding cellular biomolecules against oxidative damage (Truong et al., 2018). Dietary RES plus HMB supplementation enhanced the concentration of GSH-Px and T-AOC, while RES or HMB supplementation alone did not affect the above two indexes in the jejunum (Ji et al., 2024). In the current study, we observed that dietary RES plus HMB supplementation enhanced the concentration of GSH-Px and T-AOC, which indicated that only when RES and HMB supplemented together can they enhance the intestinal antioxidant capacity. The host immunity system, which encompasses both the innate and adaptive immune systems, serves as a crucial defense mechanism against the invasion of pathogens (Guo et al., 2015) The supplementation of a dry suspension containing resveratrol was observed to stimulate the proliferation of peripheral blood and splenic lymphocytes. Additionally, it promoted the production of IgG, regulated the release of IFN-γ, and downregulated the release of TNF-α (Fu et al., 2018). In the present study, we observed that RES supplementation enhanced the IgG level and decreased the IL-1β level, while the concentration of IL-6 elevated in the duodenum, these results suggest that dietary RES stimulates the innate and adaptive immune systems to suppress inflammatory response.

Dietary RES has been shown to influence the gut microbiota of piglets. Specifically, the RES increased the abundance of beneficial bacteria, such as *Lactobacillus* and *Alloprevotella*, while concurrently decreasing the abundance of harmful bacteria, like *Escherichia-shigella* in the piglet (Zhao et al., 2022). This indicates that RES plays a key role in preventing the excessive proliferation of harmful bacteria, thus maintaining the balance of gut microbiota and preserving bacterial diversity. In broiler chickens, dietary HMB supplementation enhanced the ACE and Shannon indexes in the cecal microbiota (Zhang et al., 2022). Dietary supplementing RES or HMB raised the indexes of alpha diversity including ACE and Chao1 of the duodenal microbiota in Tibetan sheep in the present study, suggesting that RES and HMB exert the effect of enhancing the diversity index of gut microbiota. The results of LEfSe showed that the RES group characterized by a higher relative abundance of *Butyrivibrio* in the duodenum of the current study, which is consistent with a previous study that found supplementation with RES notably elevated the population of *Fibrobacter succinogenes*, *Ruminococcus albus*, and *Butyrivibrio fibrisolvens* in sheep (Ma et al., 2015). Genus *Butyrivibrio*, plays an important role in the ruminal fermentation of polysaccharides involved in cellulose degradation to produce butyric acid (Rodríguez Hernáez et al., 2018). It is reported that butyrate, functioning as an anti-inflammatory agent, plays an essential role in modulating the immune response and intestinal barrier function of animals (Tan et al., 2014). Consequently, the increase in the relative abundance of *Butyrivibrio* may contribute to the production of more butyric acid to regulate duodenal immunity when RES is supplemented in Tibetan sheep. Genus *Rummeliibacillus* is a potential probiotic in animals, which can be used as a feed additive to enhance growth and health status (Tan et al., 2019). *UCG-002*, a member of the *Oscillospiraceae* family, has been found to be positively associated with higher growth performance in growing pigs (Ramsay et al., 2022). In addition, *UCG-002* has also been shown to enhance the antioxidant and anti-inflammatory capacity in pregnant sows (Chen et al., 2024). In this study, HMB supplementation caused a higher relative abundance of *Rummeliibacillus* and *UCG-002* in the duodenal microbiota, which may contribute to enhancing intestinal antioxidant and anti-inflammatory capacity in Tibetan sheep. However, the harmful bacteria (*Myroides* and *Pseudomonas*) had a higher abundance in the CON group. In detail, *Myroides* strains usually behave as low-grade opportunistic pathogens, and have been found to be responsible for cases of urinary tract infection, endocarditis, and ventriculitis (Benedetti et al., 2011). *Pseudomonas* has pathogenic properties that cytotoxins ExoV and ExoS are associated with intracellular membrane destruction, promotion of Rac1 inactivation and induction of necrosis and apoptosis (Kuugbee et al., 2016). Therefore, these results indicate that dietary RES or HMB supplementation promotes the abundance of beneficial bacteria, and improves the antioxidant and anti-inflammatory capacity in the duodenum in Tibetan sheep.

Except for intestinal microbiota, the current study investigated the effects of RES and HMB supplementation on the alternations of the metabolome in duodenal digesta. The results showed a total of 575 differential metabolites were identified among 4 groups. Among these differential metabolites, only 15 of them were upregulated, and downregulated metabolites enriched in protein digestion and absorption, mineral absorption, glycine, serine and threonine metabolism, lysine degradation, taste transduction, biosynthesis of amino acids, phenylalanine metabolism, monobactam biosynthesis and pyrimidine metabolism, which suggests that RES and HMB supplementation may have a negative influence on protein absorption and amino acids metabolism. These results were in contradiction with the biological function of RES and HMB. To further investigate the reason for the above results, we employed the WGCNA analysis to calculate the relationship between phenotype and metabolomics profile and obtained 2 key modules (M6 and M9), in which we obtained 37 differential metabolites in the M6 module that may affect phenotype. L-aspartic acid may be crucial in causing changes in both the luminal and extraluminal structures of the intestines, and it can also lead to intestinal fibrosis (Li et al., 2024). Gibberellic acid induced liver and kidney injury and caused oxidative stress by elevating serum MDA levels (Soliman et al., 2022). Phosphoric acid is mainly involved in oxidative phosphorylation, a process that drives ATP production and energy release from the catabolism of amino acids and others (Zhang et al., 2024). These differential metabolites co-existed of RES vs. CON, HMB vs. CON and RES_HMB vs. CON, and their concentrations were reduced, suggesting that RES, HMB and RES_HMB supplementation downregulated the above metabolites to maintain intestinal health. Furthermore, differential metabolite L-glutathione (reduced) exhibited higher level in the HMB group when compared with CON group, which are in agreement with the results of previous studies that stated diets supplemented with reduced glutathione promoted the growth performance, antioxidant capacity, the jejunal mucosa morphology and barrier function of weaned piglets (Tian et al., 2025). It is suggested that dietary HMB may enhance the antioxidant capacity of the intestine in Tibetan sheep through changing the levels of metabolites.

## 5 Conclusion

Our findings highlight that dietary RES improved trypsin secretion, villus development, antioxidant capacity, and inflammatory response in the duodenum by increasing the relative abundance of *Butyrivibrio*. Meanwhile, HMB supplementation promoted the concentrations of trypsin and cellulase, villus height, V/C ratio, and antioxidant capacity by increasing the relative abundance of *Rummeliibacillus* and *UCG_002* and increasing the concentration of L-glutathione. Supplementation with RES and HMB had positive effects on duodenal function in Tibetan sheep; however, the safety of these additives requires further validation through comprehensive toxicity and dose–response studies. The current study demonstrated beneficial effects of RES and HMB on intestinal health parameters; therefore, RES and HMB can be used as feed additives in sheep feeding to maintain intestinal health and function.

## Data Availability

The datasets presented in this study can be found in online repositories. The 16s rDNA sequence data of this study have been submitted to the Sequence Read Archive (SRA) database, and the data are accessible through SRA Series accession number PRJNA1202731. Metabolome data have been uploaded to OMIX, China National Center for Bioinformation/Beijing Institute of Genomics, Chinese Academy of Sciences with accession number OMIX010577.

## Supplementary material

FIGURE S1 Rarefaction curves of the duodenal digesta based on 16S rDNA sequencing.

FIGURE S2 The relative abundances of the main bacteria on the phylum level.

## References

[ref1] Benedetti P. Rassu M. Pavan G. Sefton A. Pellizzer G. (2011). Septic shock, pneumonia, and soft tissue infection due to *Myroides odoratimimus*: report of a case and review of Myroides infections. Infection 39, 161–165. doi: 10.1007/s15010-010-0077-1, PMID: 21246247

[ref2] Bolyen E. Rideout J. R. Dillon M. R. Bokulich N. A. Abnet C. C. Al-Ghalith G. A.. (2019). Reproducible, interactive, scalable and extensible microbiome data science using QIIME 2. Nat. Biotechnol. 37, 852–857. doi: 10.1038/s41587-019-0209-9, PMID: 31341288 PMC7015180

[ref3] Bylesjo M. Rantalainen M. Cloarec O. Nicholson J. K. Holmes E. Trygg J. (2006). OPLS discriminant analysis: combining the strengths of PLS-DA and SIMCA classification. J. Chemom. 20, 341–351. doi: 10.1002/cem.1006, PMID: 40512761

[ref4] Callahan B. J. McMurdie P. J. Rosen M. J. Han A. W. Johnson A. J. Holmes S. P. (2016). DADA2: high-resolution sample inference from Illumina amplicon data. Nat. Methods 13, 581–583. doi: 10.1038/nmeth.3869, PMID: 27214047 PMC4927377

[ref5] Chaplin A. Carpéné C. Mercader J. (2018). Resveratrol, metabolic syndrome, and gut microbiota. Nutrients 10:1651. doi: 10.3390/nu10111651, PMID: 30400297 PMC6266067

[ref6] Chen D. Chen X. Tu Y. Wang B. Lou C. Ma T.. (2015). Effects of mulberry leaf flavonoid and resveratrol on methane emission and nutrient digestion in sheep. Anim. Nutr. 1, 362–367. doi: 10.1016/j.aninu.2015.12.008, PMID: 29767046 PMC5940990

[ref7] Chen X. Zeng Z. Huang Z. Chen D. He J. Chen H.. (2021). Effects of dietary resveratrol supplementation on immunity, antioxidative capacity and intestinal barrier function in weaning piglets. Anim. Biotechnol. 32, 240–245. doi: 10.1080/10495398.2019.1683022, PMID: 31645181

[ref8] Chen M. Zhao Y. Li S. Chang Z. Liu H. Zhang D.. (2024). Maternal malic acid may ameliorate oxidative stress and inflammation in sows through modulating gut microbiota and host metabolic profiles during late pregnancy. Antioxidants (Basel) 13:253. doi: 10.3390/antiox13020253, PMID: 38397851 PMC10886295

[ref9] Douglas G. M. Maffei V. J. Zaneveld J. R. Yurgel S. N. Brown J. R. Taylor C. M.. (2020). PICRUSt2 for prediction of metagenome functions. Nat. Biotechnol. 38, 685–688. doi: 10.1038/s41587-020-0548-6, PMID: 32483366 PMC7365738

[ref10] Duan Y. Li F. Li Y. Tang Y. Kong X. Feng Z.. (2016). The role of leucine and its metabolites in protein and energy metabolism. Amino Acids 48, 41–51. doi: 10.1007/s00726-015-2067-1, PMID: 26255285

[ref7001] Fan W. Shi J. Wang B. Zhang M. Kong M. Li W. (2022). Effects of zinc and *Bacillus subtilis* on the reproductive performance, egg quality, nutrient digestion, intestinal morphology, and serum antioxidant capacity of geese breeders. Poultry Sci. 101:101677. doi: 10.1016/j.psj.2021.101677 35051674 PMC8883061

[ref11] Fu Q. Cui Q. Yang Y. Zhao X. Song X. Wang G.. (2018). Effect of resveratrol dry suspension on immune function of piglets. Evid. Based Complement. Alternat. Med. 2018:5952707. doi: 10.1155/2018/5952707, PMID: 29483932 PMC5816855

[ref12] Gan J. Ji Q. Su Q. Hou S. Gui L. (2024). Resveratrol and β-hydroxy-β-methylbutyric acid supplementation promotes ileal development and digestive function by altering microbial community abundance and metabolites in Tibetan sheep. Front. Vet. Sci. 11:1470992. doi: 10.3389/fvets.2024.1470992, PMID: 39723186 PMC11668758

[ref13] Guo L. Huang Y. Chen X. Hu-Li J. Urban J. F. Jr. Paul W. E. (2015). Innate immunological function of TH2 cells *in vivo*. Nat. Immunol. 16, 1051–1059. doi: 10.1038/ni.3244, PMID: 26322482 PMC4575627

[ref14] Hall M. Beiko R. G. (2018). 16S rRNA gene analysis with QIIME2. Methods Mol. Biol. 1849, 113–129. doi: 10.1007/978-1-4939-8728-3_8, PMID: 30298251

[ref15] Han B. Tian D. Li X. Liu S. Tian F. Liu D.. (2024). Multiomics analyses provide new insight into genetic variation of reproductive adaptability in Tibetan sheep. Mol. Biol. Evol. 41:msae058. doi: 10.1093/molbev/msae058, PMID: 38552245 PMC10980521

[ref16] Holeček M. (2017). Beta-hydroxy-beta-methylbutyrate supplementation and skeletal muscle in healthy and muscle-wasting conditions. J. Cachexia. Sarcopenia Muscle 8, 529–541. doi: 10.1002/jcsm.12208, PMID: 28493406 PMC5566641

[ref17] Ji Q. Zhang F. Zhang Y. Su Q. He T. Hou S.. (2024). Multi-omics revealed resveratrol and β-Hydroxy-β-methyl butyric acid alone or in combination improved the Jejunal function in Tibetan sheep. Antioxidants (Basel) 13:892. doi: 10.3390/antiox13080892, PMID: 39199138 PMC11351831

[ref18] Kanehisa M. Goto S. (2000). KEGG: Kyoto encyclopedia of genes and genomes. Nucleic Acids Res. 28, 27–30. doi: 10.1093/nar/28.1.27, PMID: 10592173 PMC102409

[ref19] Karl J. P. Hatch A. M. Arcidiacono S. Pearce S. C. Pantoja-Feliciano I. G. Doherty L. A.. (2018). Effects of psychological, environmental and physical stressors on the gut microbiota. Front. Microbiol. 9:2013. doi: 10.3389/fmicb.2018.02013 30258412 PMC6143810

[ref20] Kuugbee E. D. Shang X. Gamallat Y. Bamba D. Awadasseid A. Suliman M. A.. (2016). Structural change in microbiota by a probiotic cocktail enhances the gut barrier and reduces Cancer via TLR2 signaling in a rat model of Colon Cancer. Dig. Dis. Sci. 61, 2908–2920. doi: 10.1007/s10620-016-4238-7, PMID: 27384052

[ref21] Li X. Hu S. Shen X. Zhang R. Liu C. Xiao L.. (2024). Multiomics reveals microbial metabolites as key actors in intestinal fibrosis in Crohn's disease. EMBO Mol. Med. 16, 2427–2449. doi: 10.1038/s44321-024-00129-8, PMID: 39271960 PMC11473649

[ref22] Ma T. Chen D. D. Tu Y. Zhang N. F. Si B. W. Deng K. D.. (2015). Effect of dietary supplementation with resveratrol on nutrient digestibility, methanogenesis and ruminal microbial flora in sheep. J. Anim. Physiol. Anim. Nutr. (Berl.) 99, 676–683. doi: 10.1111/jpn.12264, PMID: 25319536

[ref23] Magoč T. Salzberg S. L. (2011). FLASH: fast length adjustment of short reads to improve genome assemblies. Bioinformatics 27, 2957–2963. doi: 10.1093/bioinformatics/btr507, PMID: 21903629 PMC3198573

[ref24] Meng Q. Li J. Wang C. Shan A. (2023). Biological function of resveratrol and its application in animal production: a review. J. Anim. Sci. Biotechnol. 14:25. doi: 10.1186/s40104-022-00822-z, PMID: 36765425 PMC9921422

[ref25] Mir P. Mir Z. Shaner A. Sorensen B. (1987). Nutritional performance and intestinal absorptive capacities of neonatal lambs fed milk replacer or dams milk, with or without access to creep feed. Can. J. Anim. Sci. 67, 83–91. doi: 10.4141/cjas87-010

[ref26] Moharrery A. Larsen M. Weisbjerg M. R. (2014). Starch digestion in the rumen, small intestine, and hind gut of dairy cows – a meta-analysis. Anim. Feed Sci. Technol. 192, 1–14. doi: 10.1016/j.anifeedsci.2014.03.001

[ref27] Ogata H. Goto S. Sato K. Fujibuchi W. Bono H. Kanehisa M. (1999). KEGG: Kyoto encyclopedia of genes and genomes. Nucleic Acids Res. 27, 29–34. doi: 10.1093/nar/27.1.29, PMID: 9847135 PMC148090

[ref7002] Parks D. H. Tyson G. W. Hugenholtz P. Beiko R. G. (2014). STAMP: statistical analysis of taxonomic and functional profiles. Bioinformatics. 30, 3123–3124. doi: 10.1093/bioinformatics/btu494 25061070 PMC4609014

[ref28] Plaizier J. C. Danesh Mesgaran M. Derakhshani H. Golder H. Khafipour E. Kleen J. L.. (2018). Review: enhancing gastrointestinal health in dairy cows. Animal 12, s399–s418. doi: 10.1017/s1751731118001921, PMID: 30139397

[ref29] Quast C. Pruesse E. Yilmaz P. Gerken J. Schweer T. Yarza P.. (2013). The SILVA ribosomal RNA gene database project: improved data processing and web-based tools. Nucleic Acids Res. 41, D590–D596. doi: 10.1093/nar/gks1219 23193283 PMC3531112

[ref30] Ramsay T. G. Arfken A. M. Summers K. L. (2022). Enteroendocrine peptides, growth, and the microbiome during the porcine weaning transition. Anim. Microbiome 4:56. doi: 10.1186/s42523-022-00206-8, PMID: 36401290 PMC9673406

[ref31] Rodríguez Hernáez J. Cerón Cucchi M. E. Cravero S. Martinez M. C. Gonzalez S. Puebla A.. (2018). The first complete genomic structure of Butyrivibrio fibrisolvens and its chromid. Microb. Genom. 4:e000216. doi: 10.1099/mgen.0.000216, PMID: 30216146 PMC6249431

[ref32] Rognes T. Flouri T. Nichols B. Quince C. Mahé F. (2016). VSEARCH: a versatile open source tool for metagenomics. PeerJ 4:e2584. doi: 10.7717/peerj.2584, PMID: 27781170 PMC5075697

[ref33] Rout P. K. Behera B. K. (2021). “Goat and sheep farming” in Sustainability in Ruminant Livestock.

[ref34] Shames B. (2019). “Anatomy and physiology of the duodenum” in Shackelford's surgery of the alimentary tract, vol. 2, 786–803.

[ref35] Shao Y. Wang Y. Yuan Y. Xie Y. (2021). A systematic review on antibiotics misuse in livestock and aquaculture and regulation implications in China. Sci. Total Environ. 798:149205. doi: 10.1016/j.scitotenv.2021.149205, PMID: 34375247

[ref36] Soliman M. M. Gaber A. Alsanie W. F. Mohamed W. A. Metwally M. M. M. Abdelhadi A. A.. (2022). Gibberellic acid-induced Hepatorenal dysfunction and oxidative stress: mitigation by quercetin through modulation of antioxidant, anti-inflammatory, and Antiapoptotic activities. J. Food Biochem. 46:e14069. doi: 10.1111/jfbc.14069, PMID: 34984688

[ref37] Soriano B. Paas W. Reidsma P. Martin C. S. Kopainsky B. Herrera H. (2024). Overcoming collapse of farming systems: shifting from vicious to virtuous circles in the extensive sheep farming system in Huesca (Spain). Ecol. Soc. 29:37. doi: 10.5751/es-15717-290437

[ref38] Tan H. Y. Chen S.-W. Hu S.-Y. (2019). Improvements in the growth performance, immunity, disease resistance, and gut microbiota by the probiotic *Rummeliibacillus Stabekisii* in Nile Tilapia (oreochromis Niloticus). Fish Shellfish Immunol. 92, 265–275. doi: 10.1016/j.fsi.2019.06.027, PMID: 31202962

[ref39] Tan J. McKenzie C. Potamitis M. Thorburn A. N. Mackay C. R. Macia L. (2014). The role of short-chain fatty acids in health and disease. Adv. Immunol. 121, 91–119. doi: 10.1016/b978-0-12-800100-4.00003-9 24388214

[ref40] Tian Z. Cui Y. Yu M. Deng D. Li Z. Ma X.. (2025). Reduced glutathione promoted growth performance by improving the Jejunal barrier, antioxidant function, and altering proteomics of weaned piglets. Antioxidants 14:107. doi: 10.3390/antiox14010107, PMID: 39857441 PMC11761254

[ref41] Tian B. Liu J. (2019). Resveratrol: a review of plant sources, synthesis, stability, modification and food application. J. Sci. Food Agric. 100, 1392–1404. doi: 10.1002/jsfa.10152, PMID: 31756276

[ref42] Tomasello G. Mazzola M. Leone A. Sinagra E. Zummo G. Farina F.. (2016). Nutrition, oxidative stress and intestinal dysbiosis: influence of diet on gut microbiota in inflammatory bowel diseases. Biomed. Pap. Med. Fac. Univ. Palacky Olomouc Czech Repub. 160, 461–466. doi: 10.5507/bp.2016.052, PMID: 27812084

[ref43] Tomaszewska E. Dobrowolski P. Prost L. Chand D. K. P. Donaldson J. Winiarczyk D.. (2023). The effect of supplementation with Β-hydroxy-β-methylbutyric acid (HMB) to pregnant sows on the mucosal structure, immunolocalization of intestinal barrier proteins, VIP and leptin in the large intestine in their offspring. Ann. Anim. Sci. 23, 87–96. doi: 10.2478/aoas-2021-0079

[ref44] Truong V. L. Jun M. Jeong W. S. (2018). Role of resveratrol in regulation of cellular defense systems against oxidative stress. Biofactors 44, 36–49. doi: 10.1002/biof.1399, PMID: 29193412

[ref45] Tufekci H. Sejian V. (2023). Stress factors and their effects on productivity in sheep. Animals 13:2769. doi: 10.3390/ani13172769, PMID: 37685033 PMC10486368

[ref46] Uni Z. (2006). “Early development of small intestinal function” in Avian gut function in health and disease, 29–42.

[ref47] Vancamelbeke M. Vermeire S. (2017). The intestinal barrier: a fundamental role in health and disease. Expert Rev. Gastroenterol. Hepatol. 11, 821–834. doi: 10.1080/17474124.2017.1343143, PMID: 28650209 PMC6104804

[ref48] Wang F. Xie B. Ji H. Xia J. Hao Y. Cao Z.. (2025). Temporal modulation of duodenal microbiota in dairy cows: effects of dietary shift from high forage to high concentration. Front. Vet. Sci. 12:1551327. doi: 10.3389/fvets.2025.1551327, PMID: 40256605 PMC12006166

[ref49] Yang M. Xie Q. Wang J. Zha A. Chen J. Jiang Q.. (2025). Ningxiang pig-derived *lactobacillus reuteri* modulates host intramuscular fat deposition via branched-chain amino acid metabolism. Microbiome 13:32. doi: 10.1186/s40168-024-02013-6, PMID: 39891238 PMC11786426

[ref50] Zhang H. Z. Chen D. W. He J. Zheng P. Yu J. Mao X. B.. (2019). Long-term dietary resveratrol supplementation decreased serum lipids levels, improved intramuscular fat content, and changed the expression of several lipid metabolism-related Mirnas and genes in growing-finishing Pigs1. J. Anim. Sci. 97, 1745–1756. doi: 10.1093/jas/skz057, PMID: 30852606 PMC6447270

[ref51] Zhang X. Han L. Hou S. Raza S. H. A. Gui L. Sun S.. (2022). Metabolomics approach reveals high energy diet improves the quality and enhances the flavor of black Tibetan sheep meat by altering the composition of rumen microbiota. Front. Nutr. 9:915558. doi: 10.3389/fnut.2022.915558, PMID: 36034898 PMC9405419

[ref52] Zhang R. Zhang W. B. Bi Y. L. Tu Y. Ma T. Dong L. F.. (2019). Sanguinarine and resveratrol affected rumen fermentation parameters and bacterial community in calves. Anim. Feed Sci. Technol. 251, 64–75. doi: 10.1016/j.anifeedsci.2019.03.004

[ref53] Zhang F. Zhang Y. He T. Ji Q. Hou S. Gui L. (2024). Changes in rumen microbiology and metabolism of Tibetan sheep with different Lys/met ratios in low-protein diets. Animals 14:1533. doi: 10.3390/ani14111533, PMID: 38891581 PMC11171176

[ref54] Zhao Y. Huang Y. Gao K. Wen X. Hu S. Wang L.. (2022). Maternal resveratrol regulates the growth performance, antioxidant capacity, and intestinal health of suckling piglets through intestinal microorganisms at high summer temperatures. Front. Nutr. 9:971496. doi: 10.3389/fnut.2022.971496, PMID: 36159472 PMC9501877

[ref55] Zheng C. Song B. Duan Y. Zhong Y. Yan Z. Zhang S.. (2020). Dietary Β-Hydroxy-β-methylbutyrate improves intestinal function in weaned piglets after lipopolysaccharide challenge. Nutrition 78:110839. doi: 10.1016/j.nut.2020.110839, PMID: 32540677

