# Supplementary figures and images for: The influence of resveratrol and β-Hydroxy-β-methyl butyric acid supplementation alone or in combination on the development and health of the duodenum in Tibetan sheep
Source: Front Microbiol. 2025 Jul 8;16:1612102. doi: 10.3389/fmicb.2025.1612102 (PMC12279740; doi:10.3389/fmicb.2025.1612102)

# Multi Samples Rarefaction Curves

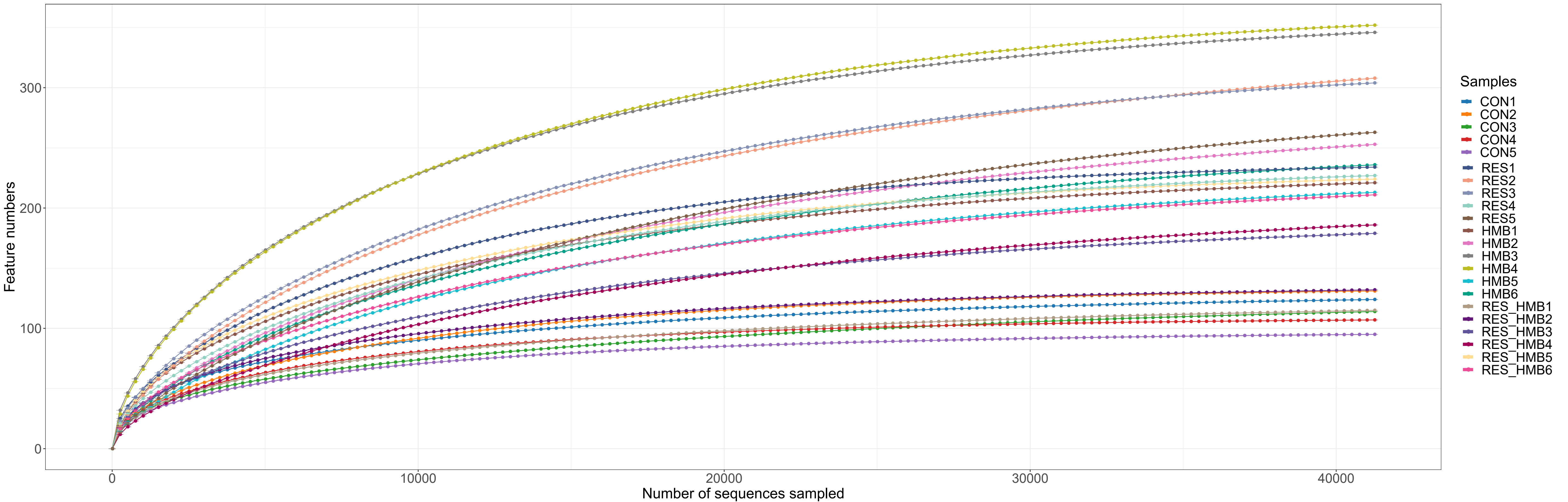

Supplement: FIGURE S1 — Rarefaction curves of the duodenal digesta based on 16S rDNA sequencing. [file Data_Sheet_1.pdf]

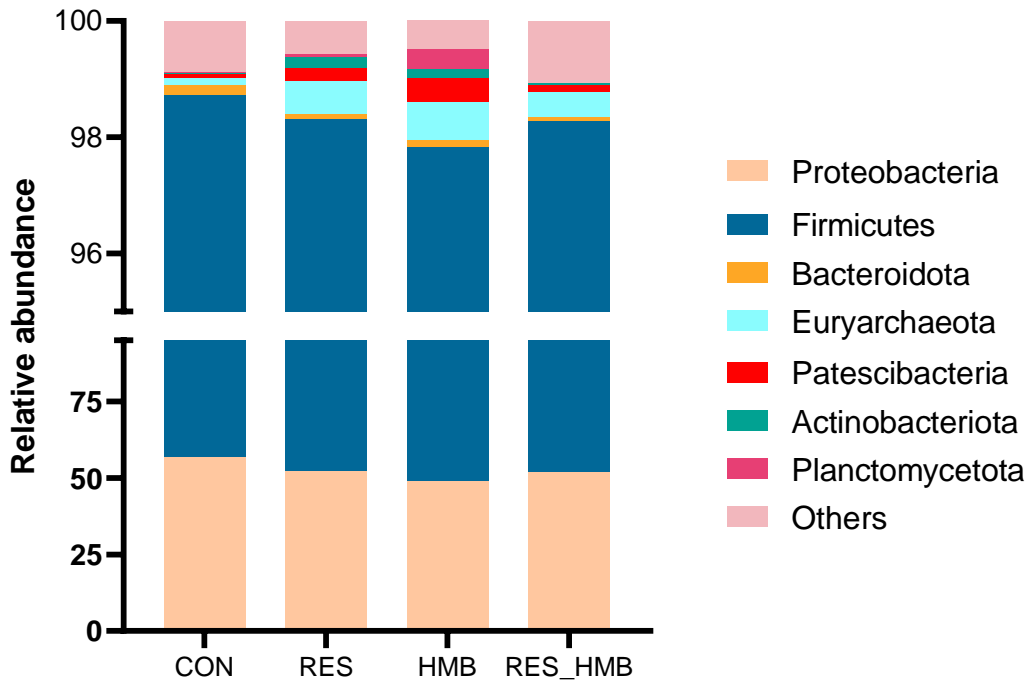

Supplement: FIGURE S2 — The relative abundances of the main bacteria on the phylum level. [file Data_Sheet_2.pdf]
